# A Canadian Experience With Off-the-Shelf, Aseptically Processed, Costal Cartilage Segment Allografts in Complex Rhinoplasty

**DOI:** 10.1093/asjof/ojac085

**Published:** 2022-11-22

**Authors:** John Milkovich, Jamil Ahmad

**Affiliations:** Student, Faculty of Engineering, McMaster University, Hamilton, Ontario, Canada; Director of education and research at a private plastic surgery practice, Mississauga, Ontario, Canada

## Abstract

**Background:**

Complex primary and secondary rhinoplasties usually necessitate grafting materials when native nasal cartilage is inadequate for reconstruction. Fresh frozen, aseptically processed, and nonterminally sterilized costal cartilage segment allografts (CCSAs) are a novel grafting material for such cases that avoid donor-site morbidity, improve operating efficiency, and mitigate the postoperative risks.

**Objectives:**

To report the early experience using fresh frozen, aseptically processed, and nonterminally sterilized CCSAs used in complex primary and secondary rhinoplasties, in Canada.

**Methods:**

We retrospectively reviewed 21 patients (17 female and 4 male patients) who underwent a primary or secondary rhinoplasty surgery using CCSAs from June 2019 to April 2022.

**Results:**

The mean age was 39 years (range, 27-58 years), and the mean body mass index was 23.7 kg/m^2^ (range, 24-40 kg/m^2^). Of the 21 procedures, 11 were primary (52.4%) and 10 were secondary (47.6%) rhinoplasties. The mean operative time was 185 min (range, 85-330 min), with a mean follow-up time of 15.0 months (range, 2.0-37.8 months). At follow-up, 19 patients (90.5%) reported being “very satisfied” with their aesthetic results, and only 2 (9.5%) underwent revision surgery. No serious complications were reported, and only 1 case showed evidence of graft resorption.

**Conclusions:**

Based on early experience, this CCSA avoids donor-site morbidity and reduces operative time while maintaining a low complication rate, providing a viable alternative to the use of autologous costal cartilage when indicated in complex primary or secondary rhinoplasties with inadequate native nasal cartilage.

**Level of Evidence: 4:**

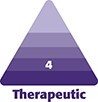

Management of complex primary and secondary rhinoplasty cases is challenging. Reconstruction of the nasal osseocartilaginous framework often requires a substantial amount of cartilage, necessitating the use of grafting materials when there is inadequate native nasal cartilage. Autologous cartilage grafts are often preferred in these instances because of their low rates of infection and extrusion compared to alloplastic materials. Each autologous cartilage graft donor site—nasal septum, ear, and rib—offers distinct advantages and disadvantages that must be carefully weighed by the surgeon and the patient. The optimal source of cartilage grafts would minimize the risks of resorption, infection, and warping while limiting donor-site morbidity.^1,2^

The advent of terminally sterilized allografts has created a paradigm shift in grafting materials that avoids donor-site morbidity.^3^ It is important to note, however, that terminal sterilization involves treating individually processed grafts with a high dosage of gamma radiation (usually >25 KgY). Compared to autologous costal graft cartilage, terminally sterilized homologous costal cartilage has a significantly higher resorption rate.^4^

To avoid donor-site morbidity and mitigate the risks of postoperative complications, fresh frozen, aseptically processed, and nonterminally sterilized costal cartilage segment allografts (CCSAs) have emerged, which are readily available, and have demonstrated safety and efficacy in several studies.^2,5,6^ This study reports the early experience of a single surgeon performing complex primary and secondary rhinoplasty procedures using fresh frozen, aseptically processed, and nonterminally sterilized CCSAs, in Canada.

## METHODS

### Study Design and Sample

A retrospective chart review was performed in all patients who underwent rhinoplasty using CCSA by a single surgeon in Canada. Written consent was provided, by which the patients agreed to the use and analysis of their data. This study was approved by the WCG IRB Connexus, and the ethical standards of the Declaration of Helsinki were adhered to.

### Data Collection Methods

All patients who received fresh frozen, aseptically processed, and nonterminally sterilized CCSAs produced by MTF Biologics (Profile, MTF, Edison, NJ) during primary or secondary rhinoplasty surgery, between June 2019 and April 2022, were included. These CCSAs are intended to be used when there is inadequate cartilage from the patient's nasal septum. Demographic and procedural characteristics were reviewed for each patient.

### Allograft Treatment and Properties

MTF applies strict donor screening criteria and utilizes a unique aseptic process and sterilization technique for its rib cartilage allografts that avoid terminal sterilization and meet USP 〈71〉 Sterility Tests.^7^ Donors must be <55 years old, test negative for human immunodeficiency virus and hepatitis B and C, and must not have sepsis or active malignancy. CCSAs are recovered from the donors' ribs, aseptically processed and packaged, and kept frozen.

Subsequent treatment involves debridement of excess soft tissue and trimming the costal cartilage allograft to an appropriate segment shape and size. The allografts are rinsed with a light surfactant to separate blood, lipid, and cellular components from the donated tissue and treated with an antibiotic solution to disinfect them of any pathogens or their remnants. Once these preparations are complete, the costal cartilage is rinsed again and aseptically packaged under strictly controlled conditions. Representative sample specimens undergo sensitive tests for microbes before being sealed and frozen at temperatures ranging from −40°C to −80°C. These cold conditions are sustained during transport with dry ice, and the CCSAs are thawed to room temperature prior to safe implantation.^2^

### Operative Technique and Perioperative Care

**Figure 1.**
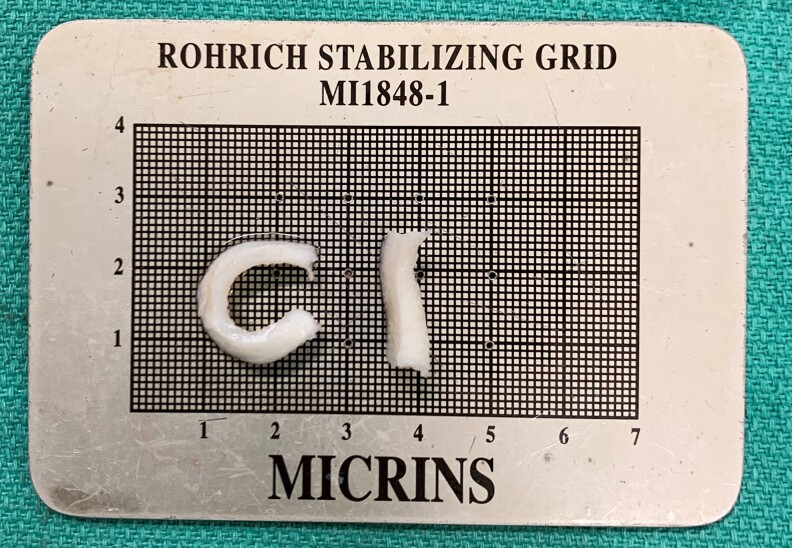
It is important to carve the segments in the correct orientation using more central sheets which have less potential to warp. Peripheral sheets have much more tendency toward warping. With reference to the native anatomic position, the costal cartilage segment should be carved into sheets with cuts made in the anterior-posterior orientation as opposed to the cranial-caudal orientation. The piece of cartilage on the left was carved from the periphery of the rib in the cranial-caudal orientation, while the piece of cartilage on the right was carved from the center of the rib in the anterior-posterior orientation.

Rhinoplasty is performed under general anesthesia as a day surgery procedure.^8^ Preoperative surgical prophylactic antibiotics are given, but postoperative prophylactic antibiotics are not routinely used. The open approach with structural cartilage grafting is performed.^9^ In this study, only large Profile Costal Cartilage segments were used as opposed to precut sheets. Balanced cross sectional cartilage carving techniques were used. It is important to carve the segments in the correct orientation using more central sheets which have less potential to warp (Figure 1). Peripheral sheets have much more tendency toward warping. With reference to the native anatomic position, the CCSA should be carved into sheets with cuts made in the anterior-posterior orientation as opposed to the cranial-caudal orientation.

Postoperatively, patients have sutures, and external and internal nasal splints in place for 1 week. Patients have routine follow-up at 1, 2, 4 to 6 weeks, and after 1 year. Clinical findings and patient satisfaction data were collected at each visit.

## RESULTS

**Figure 2.**
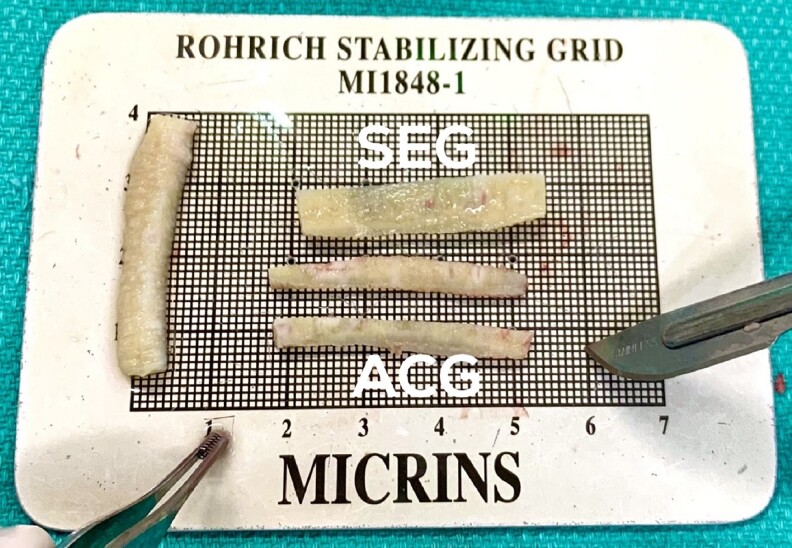
Grafts carved from the central segment of a fresh frozen, nonterminally sterilized, costal cartilage. ACG, alar contour graft; SEG, septal extension graft.

Between June 2019 and April 2022, a total of 21 patients (17 female and 4 male patients) were identified and included. The mean age was 39 years (range, 27-58 years), and the mean body mass index was 23.7 kg/m^2^. Of the 21 surgeries, 11 were primary (52.4%) and 10 were secondary (47.6%) rhinoplasties. The types of primary cases requiring CCSA included posttraumatic rhinoplasty, ethnic rhinoplasty, rhinoplasty requiring dorsal augmentation, and rhinoplasty with preexisting septal perforations. The average length of surgery was 185 min (range, 85-330 min), and the mean follow-up time was 15.0 months (range, 2.0-37.8 months). Detailed demographic information is included in Table 1. The types of grafts fashioned from the MTF allograft are outlined in Table 2 (Figure 2).

**Table 1.** Patient Demographics for Rhinoplasty With Use of a CCSA (*n* = 21)

Patient demographic/procedural characteristic	Mean (range)/no. (percent)
Age, years	39 (27-58)
Sex	Male: 4 (19) Female: 17 (81)
BMI, kg/m^2^	23.7 (17.0-32.1)
Length of surgery, minutes	185 (85-330)
Most recent follow-up, months	15.0 (2.0-37.8)
Number of previous rhinoplasties	None: 11 (52) 1 surgery: 9 (43) 2 surgeries: 1 (5)

BMI, body mass index; CCSAs, costal cartilage segment allografts; *n*, number.

**Table 2.** Graft Types Carved From CCSA

Graft type	No. of patients (%)
Columellar strut	9 (42.8%)
Septal extension	13 (61.9%)
Alar contour	16 (76.1%)
Dorsal onlay	2 (9.5%)
Extended spreader	10 (47.6%)
Splinting	7 (33.3%)
Infratip shield	2 (9.5%)
Lateral crural strut	5 (23.8%)
Diced cartilage	1 (4.8%)

CCSA, costal cartilage segment allografts.

**Figure 3.**
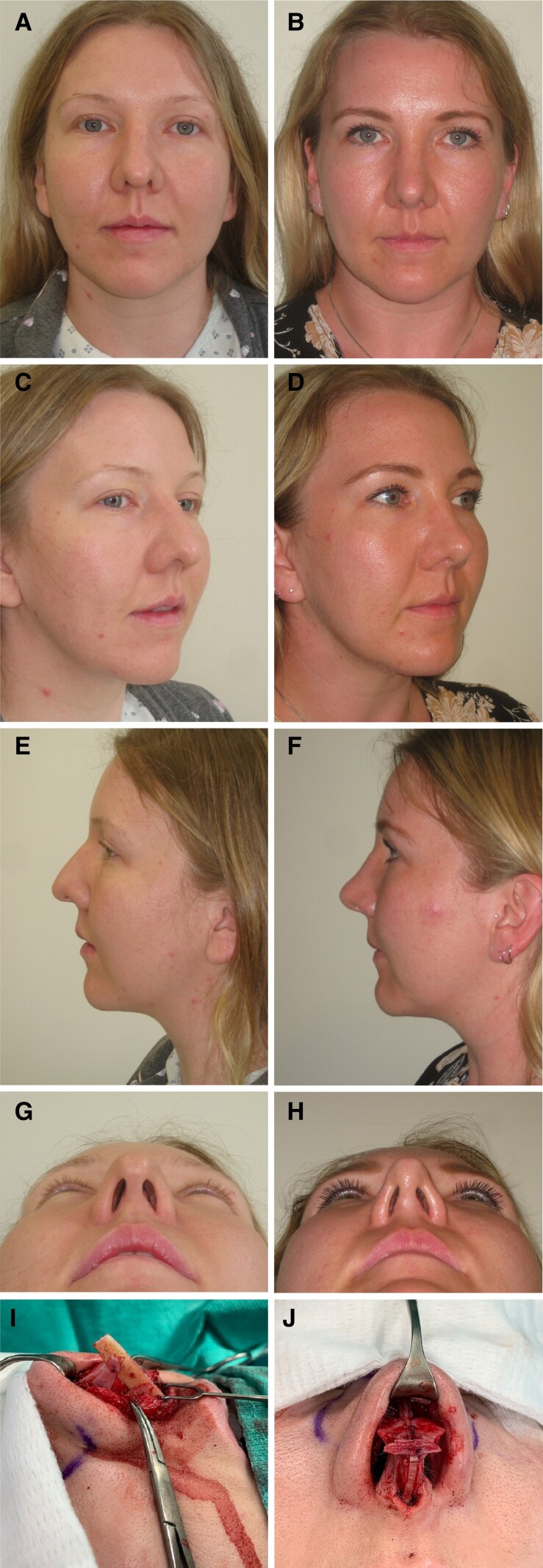
A 32-year-old female is shown (A, C, E, G) preoperatively. She has very thick skin, asymmetric dorsal aesthetic lines, and dorsal fullness and columellar and alar retraction. She has an underprojected, underrotated, asymmetric, bulbous tip. An open approach was used with component dorsal hump reduction and septal reconstruction. Bilateral osteotomies were performed. More graft material was required so costal cartilage segment allografts (CCSAs) was used for graft solution. (I) A caudal septal extension graft sutured to the septum with bilateral splinting grafts was used to lengthen the nose and increase tip projection; the splinting grafts anteriorly were from septal cartilage and posteriorly were from CCSA. The dorsum was reconstituted using bilateral autospreader flaps. The medial crura were sutured to a caudal septal extension graft. Tip shape and symmetry was achieved using a paradomal cephalic trim, and transdomal and interdomal sutures. (J) A morselized septal butterfly graft was placed across the infratip extending into the soft tissue triangles. Alar contour grafts fashioned from CCSA were placed. (B, D, F, H) The patient is shown 15 months postoperatively.

**Figure 4.**
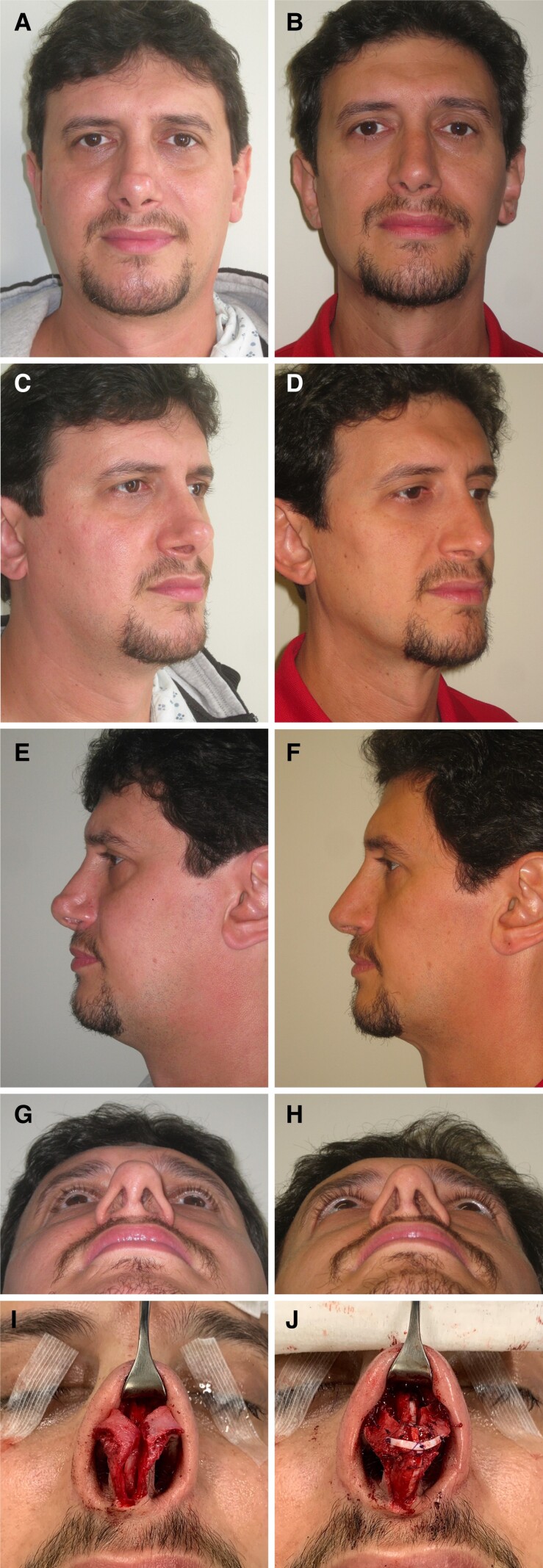
A 43-year-old male is shown (A, C, E, G) preoperatively after previous septorhinoplasty. He has thin skin, dorsal deviation to the right, and alar retraction. He has an asymmetric, boxy tip with infratip lobular excess. He also has an intranasal synechia. An open approach was used with component approach to the dorsum to release the deforming forces and allow access to the septum. The intranasal synechia was divided. There was no septum remaining for graft material, so costal cartilage segment allografts (CCSAs) were used for all grafting. The dorsum was reconstituted using a left extended spreader graft, and this was sutured to a caudal septal extension graft. The caudal septal extension graft was sutured to the caudal septum and the anterior nasal spine. Clocking sutures from the left upper lateral cartilage were sutured to the septum-spreader graft complex to further correct the rightward dorsal deviation. The right upper lateral cartilage was sutured to the dorsum with upper lateral cartilage tension spanning sutures. (I, J) The medial crura were sutured to a columellar strut graft. Tip shape and symmetry was achieved using a left paradomal cephalic trim and transdomal and interdomal sutures. A long horn graft was placed across the infratip extending into the soft tissue triangles. The lateral crura were released from the vestibular mucosa and underlay lateral crural strut grafts were placed to address the recurvature of the lateral crura. (J) Alar contour grafts were placed. (B, D, F, H) The patient is shown 2 years postoperatively.

**Figure 5.**
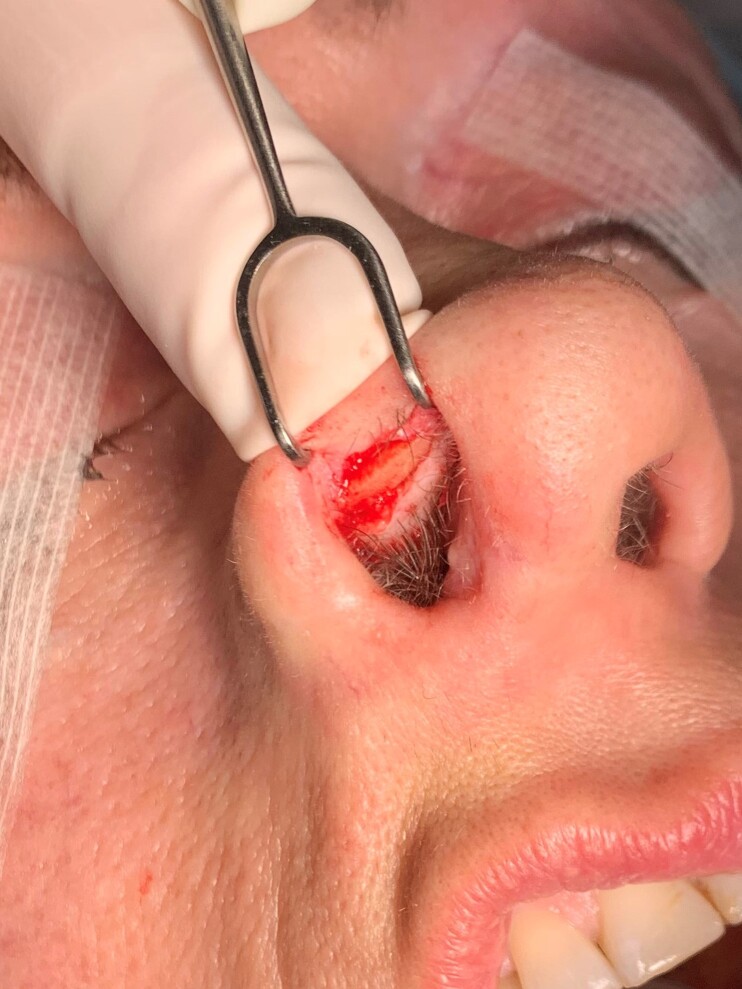
This 53-year-old female patient underwent revision rhinoplasty 1 year following secondary rhinoplasty using costal cartilage segment allografts (CCSAs). The allografts were observed to be intact during revision rhinoplasty. The right alar contour graft carved from CCSA is shown 1 year following its original placement.

Among all 21 patients, none reported any serious complications. The aesthetic outcomes were positive, and 19 patients (90.5%) reported being “very satisfied” with the results (Figures 3, 4), while 2 patients (9.5%) reported being “satisfied” but requested revision surgery to address minor aesthetic concerns. There were no infections or signs suspicious for infection with the CCSAs. At follow-up visits, there was no evidence of aesthetic deformity secondary to graft warping. Only 1 patient (4.8%) experienced some resorption when the CCSA was modified to be used as diced cartilage wrapped in autologous temporal fascia for dorsal augmentation. Two patients (9.5%) underwent revision surgery under general anesthesia to further improve the aesthetic result. At revision surgery, previously placed CCSAs were observed to be intact in both patients (Figure 5).

## DISCUSSION

The purpose of this study was to further evaluate the safety and efficacy of fresh frozen, aseptically processed, and nonterminally sterilized CCSAs in complex primary and secondary rhinoplasty cases performed in Canada. All donor tissue was sourced from the United States and used in Canada. We did not experience any difficulty with obtaining the CCSAs from the United States for use in Canada. Our early experience has been favorable, with CCSAs providing stable structural support and predictable results at short/mid-term follow-up. Although this is a small clinical series, complications were rare with no evidence of infection or warping of the CCSA, and resorption was not clinically appreciable when the CCSA was carved into sheets. However, 1 patient experienced resorption when the CCSA was diced and wrapped in autologous temporal fascia for dorsal augmentation. Two patients underwent revision surgery for further aesthetic refinement, but this was not attributed to a failure of the CCSA; these aesthetic issues were not secondary to warping or resorption of the CCSA. These findings offer further support for the use of CCSA in managing patients who lack sufficient native nasal cartilage to create a structurally stable nasal osseocartilaginous framework.

In comparison to our experience, a recent study evaluated the safety and efficacy of CCSA with a similar sample size (26 vs 21 patients) and mean follow-up time (15.9 vs 15.0 months) of patients undergoing primary or revision rhinoplasty.^10^ Importantly, Rogal et al noted a 3.6% complication rate among its cohort, which is consistent with terminally sterilized allografts^11,12^ and higher than documented rates of autologous septal and auricular cartilage grafts (<2%).^13,14^ In contrast, we reported no complications in our cohort using CCSA, which provides encouraging results compared to both terminally irradiated allografts and autologous grafts.

Furthermore, the findings of our study are in accordance with other studies supporting the use of CCSAs.^2,5,14,15^ Mohan et al^2^ reported good perioperative aesthetic outcomes, no incidences of warping and/or resorption, and only 1 incidence of infection among 50 patients. In their 9-year retrospective study, Rohrich et al^5^ noted that 2.7% (*n* = 226) of patients experienced warping and infection, while none reported displacement or extrusion of the graft.

Traditional methods for reconstructing the nasal osseocartilaginous framework with limited native nasal cartilage have included alloplastic and autogenous cartilage grafts. Alloplastic implants are useful in practice because they offer prefabrication methods, minimal absorption, no donor-site morbidity, and precise carving.^6^ However, the incidence of infection and implant extrusion is a significant disadvantage.^6,13,16^ Septal cartilage grafts are versatile and convenient to harvest, suited for both onlay and structural grafts;^12,16,17^ however, their limited supply may be insufficient for complex primary and secondary rhinoplasties, as well as reconstructive posttraumatic cases.^12,17^ Harvesting cartilage from the ear is the next best approach considering that the donor site lies in the same surgical field and leaves an inconspicuous scar;^13^ however, the cartilage's intrinsic curvature and structural frailty limit its use for specific types of grafts used to provide rigid structural support such as columellar strut grafts.^18–20^ Importantly, secondary rhinoplasties require additional support to resist the contractile forces exerted by the surrounding soft tissue caused by iatrogenic scarring. Thus, a patient's costal cartilage affords a graft with rigid structural support and is abundant in supply, which deems it more appropriate for secondary rhinoplasties where significant amounts of cartilage may be required.^21^ These benefits, however, are offset by the remote surgical site of the donor site, which poses risks of pneumothorax and lung injury,^22^ compounded by the prolongation of operative time.^23^

While these results are encouraging, this study is limited by its small sample size. Further research into the longer term structural properties and behavior of these novel allografts is also recommended.

Overall, fresh frozen, aseptically processed, and nonterminally sterilized CCSAs meet the criteria for an ideal grafting material, avoiding donor-site morbidity.^3^ Furthermore, compared with autologous costal cartilage grafts, costal cartilage allograft has a significantly higher resorption rate when terminally sterilized.^4^ Histological findings showed that the radiation reduced both the uniform distribution and size of the viable chondrocytes and the expression of proteoglycans and collagen in the extracellular matrix, thereby compromising the graft's load-bearing viscoelastic behavior and structural integrity.^4^ Along with previous studies.^2,5,10,14,15^ our study sheds a positive light on fresh frozen, aseptically processed, and nonterminally sterilized CCSAs, overcoming these limitations to produce satisfactory aesthetic outcomes.

## Conclusions

Our early experience in Canada using fresh frozen, aseptically processed, and nonterminally sterilized CCSAs for complex primary and secondary rhinoplasties when inadequate autologous cartilage is available from the nose has yielded favorable results. This allograft avoids donor-site morbidity while maintaining a low complication rate and provides an off-the-shelf alternative to the use of autologous costal cartilage.

## References

[1] Wee JH, Park MH, Oh S, Jin HR. Complications associated with autologous rib cartilage use in rhinoplasty: a meta-analysis. JAMA Facial Plast Surg. 2015;17(1):49–55. doi: 10.1001/JAMAFACIAL.2014.914 25429595

[2] Mohan R, Shanmuga Krishnan RR, Rohrich RJ. Role of fresh frozen cartilage in revision rhinoplasty. Plast Reconstr Surg. 2019;144(3):614–622. doi: 10.1097/PRS.0000000000005996 31461014

[3] Menger DJ, Nolst Trenité GJ. Irradiated homologous rib grafts in nasal reconstruction. Arch Facial Plast Surg. 2010;12(2):114–118. doi: 10.1001/ARCHFACIAL.2010.6 20231593

[4] Wee JH, Mun SJ, Na WS, et al Autologous vs irradiated homologous costal cartilage as graft material in rhinoplasty. JAMA Facial Plast Surg. 2017;19(3):183–188. doi: 10.1001/JAMAFACIAL.2016.1776 28334327 PMC5540002

[5] Rohrich RJ, Abraham J, Alleyne B, Bellamy J, Mohan R. Fresh frozen rib cartilage grafts in revision rhinoplasty: a 9-year experience. Plast Reconstr Surg. 2022;150(1):58–62. doi: 10.1097/PRS.0000000000009203 35511072

[6] Fisher M, Alba B, Ahmad J, et al Current practices in dorsal augmentation rhinoplasty. Plast Reconstr Surg. 2022;149(5):1088–1102. doi: 10.1097/PRS.0000000000009057 35259145

[7] MTF Biologics . The natural allograft solution for rhinoplasty. Accessed November 5, 2022. https://www.mtfbiologics.org/docs/default-source/product/mktg-1245_rev_3_profile_brochure_final.pdf.

[8] Rohrich RJ, Ahmad J. Rhinoplasty. Plast Reconstr Surg. 2011;128(2):49e–73e. doi: 10.1097/PRS.0B013E31821E7191 21788798

[9] Rohrich RJ, Ahmad J. A practical approach to rhinoplasty. Plast Reconstr Surg. 2016;137(4):725e–746e. doi: 10.1097/PRS.0000000000002240 27018701

[10] Rogal J, Glasgold A, Glasgold RA. Safety and efficacy of non-and minimally irradiated homologous costal cartilage in primary and revision rhinoplasty. Facial Plast Surg Aesthet Med. 2021;23(1):25–30. doi: 10.1089/fpsam.2020.0031 32522045

[11] Kridel RWH, Ashoori F, Liu ES, Hart CG. Long-term use and follow-up of irradiated homologous costal cartilage grafts in the nose. Arch Facial Plast Surg. 2009;11(6):378–394. doi: 10.1001/ARCHFACIAL.2009.91 19917899

[12] Malone M, Pearlman S. Dorsal augmentation in rhinoplasty: a survey and review. Facial Plast Surg. 2015;31(3):289–294. doi: 10.1055/S-0035-1555616 26126225

[13] Sajjadian A, Rubinstein R, Naghshineh N. Current status of grafts and implants in rhinoplasty: part I. Autologous grafts. Plast Reconstr Surg. 2010;125(2):40e–49e. doi: 10.1097/PRS.0B013E3181C82F12 19910845

[14] Rohrich RJ, Dayan E, Brito I, Gronet E, Durand PD. Warping characteristics of rib allograft cartilage. Plast Reconstr Surg. 2020;146(1):37E–42E. doi: 10.1097/PRS.0000000000006896 32590648

[15] Rohrich RJ, Shanmugakrishnan RR, Mohan R. Rhinoplasty refinements: revision rhinoplasty using fresh frozen costal cartilage allograft. Plast Reconstr Surg. 2020;145(6):1050e–1053e. doi: 10.1097/PRS.0000000000006864 32459773

[16] Sajjadian A, Naghshineh N, Rubinstein R. Current status of grafts and implants in rhinoplasty: part II. Homologous grafts and allogenic implants. Plast Reconstr Surg. 2010;125(3):99e–109e. doi: 10.1097/PRS.0B013E3181CB662F 20195087

[17] Daniel RK . Rhinoplasty: dorsal grafts and the designer dorsum. Clin Plast Surg. 2010;37(2):293–300. doi: 10.1016/J.CPS.2009.12.009 20206746

[18] Allcroft R, Friedman C, Quatela V. Cartilage grafts for head and neck augmentation and reconstruction. Autografts and homografts. Otolaryngol Clin North Am. 1994;27(1):69–80. doi: 10.1016/S0030-6665(20)30716-7 8159428

[19] Tardy ME, Denneny J, Fritsch MH. The versatile cartilage autograft in reconstruction of the nose and face. Laryngoscope. 1985;95(5):523–533. doi: 10.1288/00005537-198505000-00003 3990484

[20] Murrell GL . Auricular cartilage grafts and nasal surgery. Laryngoscope. 2004;114(12):2092–2102. doi: 10.1097/01.MLG.0000149440.20608.7C 15564827

[21] Lovice DB, Mingrone MD, Toriumi DM. Grafts and implants in rhinoplasty and nasal reconstruction. Otolaryngol Clin North Am. 1999;32(1):113–141. doi: 10.1016/S0030-6665(05)70118-3 10196441

[22] Marin VP, Landecker A, Gunter JP. Harvesting rib cartilage grafts for secondary rhinoplasty. Plast Reconstr Surg. 2008;121(4):1442–1448. doi: 10.1097/01.PRS.0000302467.24489.42 18349667

[23] Hardy KL, Davis KE, Constantine RS, et al The impact of operative time on complications after plastic surgery: a multivariate regression analysis of 1753 cases. Aesthet Surg J. 2014;34(4):614–622. doi: 10.1177/1090820X14528503/3/10.1177_1090820X14528503-FIG1.JPEG 24696297

